# Preconception mental health and the relationship between antenatal depression or anxiety and gestational diabetes mellitus: a population-based cohort study

**DOI:** 10.1186/s12884-022-05002-5

**Published:** 2022-08-31

**Authors:** Grace A. Thiele, Deirdre M. Ryan, Tim F. Oberlander, Gillian E. Hanley

**Affiliations:** 1 Department of Obstetrics & Gynaecology, University of British Columbia, Rm 590 828 West 10th Ave, Vancouver, BC V5Z 1M9 Canada; 2 Departments of Psychiatry, University of British Columbia (UBC), 938 W 28th Ave, Vancouver, BC V5Z 4H4 Canada; 3 Department of Pediatrics, University of British Columbia (UBC), 938 W 28th Ave, Vancouver, Canada

**Keywords:** Administrative data, Anxiety, Depression, Gestational diabetes mellitus, Perinatal mental health, Retrospective cohort study

## Abstract

**Background:**

Antenatal depression and anxiety are highly prevalent conditions that have been associated with increased risk for myriad adverse outcomes. Current literature exploring the connection between antenatal mental health and gestational diabetes mellitus (GDM) is limited, presenting conflicting evidence. We sought to evaluate the association between antenatal depression/anxiety (DEP-ANX) and GDM using population-based, administrative data, accounting for aspects of preconception mental health.

**Methods:**

In this population-based retrospective cohort study, we included all singleton births in British Columbia, Canada from April 1, 2000, to December 31, 2014. We identified instances of DEP-ANX from outpatient and inpatient records that included relevant diagnostic codes and stratified our cohort by preconception DEP-ANX persistence. Logistic regression models were run to estimate odds of GDM given antenatal DEP-ANX. Models were adjusted for the birthing person’s socio-demographics and pregnancy characteristics. Using an expanded cohort, we ran conditional logistic regression models that matched birthing people to themselves (in a subsequent pregnancy) based on discordance of exposure and outcome.

**Results:**

Out of the 228,144 births included in this study, 43,664 (19.1%) were to birthing people with antenatal health service use for DEP-ANX. There were 4,180 (9.6%) cases of GDM among those antenatal exposure to DEP-ANX compared to 15,102 (8.2%) among those without exposure (SMD 0.049). We observed an unadjusted odds ratio (OR) of 1.19 (95% CI: 1.15 – 1.23) and fully adjusted OR of 1.15 (95% CI: 1.11 – 1.19) overall. Apparent risk for GDM given antenatal DEP-ANX was highest among the no DEP-ANX history stratum, with a fully adjusted OR of 1.24 (95% CI: 1.15 – 1.34). Associations estimated by matched sibling analysis were non-significant (fully adjusted OR 1.19 [95% CI: 0.86 – 1.63]).

**Conclusions:**

Results from this population-based study suggest an association between antenatal DEP-ANX and GDM that varied based on mental health history. Our analysis could suggest that incident cases of DEP-ANX within pregnancy are more closely associated with GDM compared to recurring or chronic cases.

**Supplementary Information:**

The online version contains supplementary material available at 10.1186/s12884-022-05002-5.

## Introduction

Depression and anxiety are highly prevalent among individuals of reproductive age [1–3], and nearly 20% of birthing people experience an episode of depression or anxiety during pregnancy [4, 5]. Preconception mental health disorders increase risk for an antenatal episode [6–9], and both preconception and antenatal depression/anxiety have been independently associated with adverse perinatal outcomes such as gestational diabetes mellitus (GDM) [10, 11]. While the risk from preconception and antenatal mental health disorders is a critical issue, very little research has aimed to understand how different mental health trajectories are associated with adverse outcomes like GDM.

GDM is a unique subtype of diabetes mellitus (DM), indicated by glucose intolerance first detected during pregnancy. GDM has been shown to pose significant risks to perinatal health, increasing the likelihood of hemorrhage, preeclampsia, and operative delivery [12–14]. Instances of GDM have been shown to also have lasting impacts on cardiovascular and metabolic health, with elevated risk for future cardiovascular disease and type 2 DM for both the birthing person and child [15–17]. Cases of comorbid antenatal depression/anxiety (DEP-ANX) and GDM have been further shown to increase the likelihood of adverse outcomes, including preeclampsia to preterm birth [18, 19].

Current literature investigating the link between antenatal DEP-ANX and GDM is limited, and presents conflicting evidence regarding the significance and magnitude of this association [11, 20–26]. There are similarly contradictory results regarding the connection between depression/anxiety history and GDM [10, 27, 28]. Our understanding of this relationship is thus largely guided by studies focused on bidirectional associations between DEP-ANX and DM [29, 30]. DEP-ANX and DM are hypothesized to originate from shared pathways, in which trauma, genetics, environment, and inequities contribute to the activation of physiological responses driving both conditions [31, 32].

In this study, we aimed to incorporate preconception mental health into a study of how antenatal depression/anxiety influences risk for GDM. We hypothesized that compared to those without history of DEP-ANX, individuals with chronic histories would have higher odds of GDM related to antenatal DEP-ANX, given increased exposure to chronic stress and its cumulative effects (allostatic load) [33, 34].

## Materials and methods

We conducted a population-based, retrospective cohort study of all live births in British Columbia (BC), Canada from April 1, 2000, to December 31, 2013. Birthing person data were collected from 10-years preconception through delivery. Population Data (PopData) BC created our cohort through the BC Perinatal Data Registry (BCPDR) [35], containing nearly 100% of births in BC, regardless of place of delivery. They then linked these data with the Discharge Abstract Database (DAD) [36], documenting all BC hospital stays and day surgeries; the Medical Services Plan (MSP) Payment Information File [37], describing all BC medical visits; vital statistics data [38], containing birth information; and the Central Demographics File (previously BC Consolidation file) [39], detailing demographic and registration data for provincial health coverage (MSP).

Ethics approval for our use of deidentified administrative data was approved by the University of British Columbia Behavioural Research Ethics Board. Data access was approved by the Data Stewards. Both approvals include a waiver of informed consent from participants. All inferences, opinions, and conclusions drawn are those of the authors and do not reflect opinions or policies of the Data Stewards.

### Study cohort

**Fig. 1:**
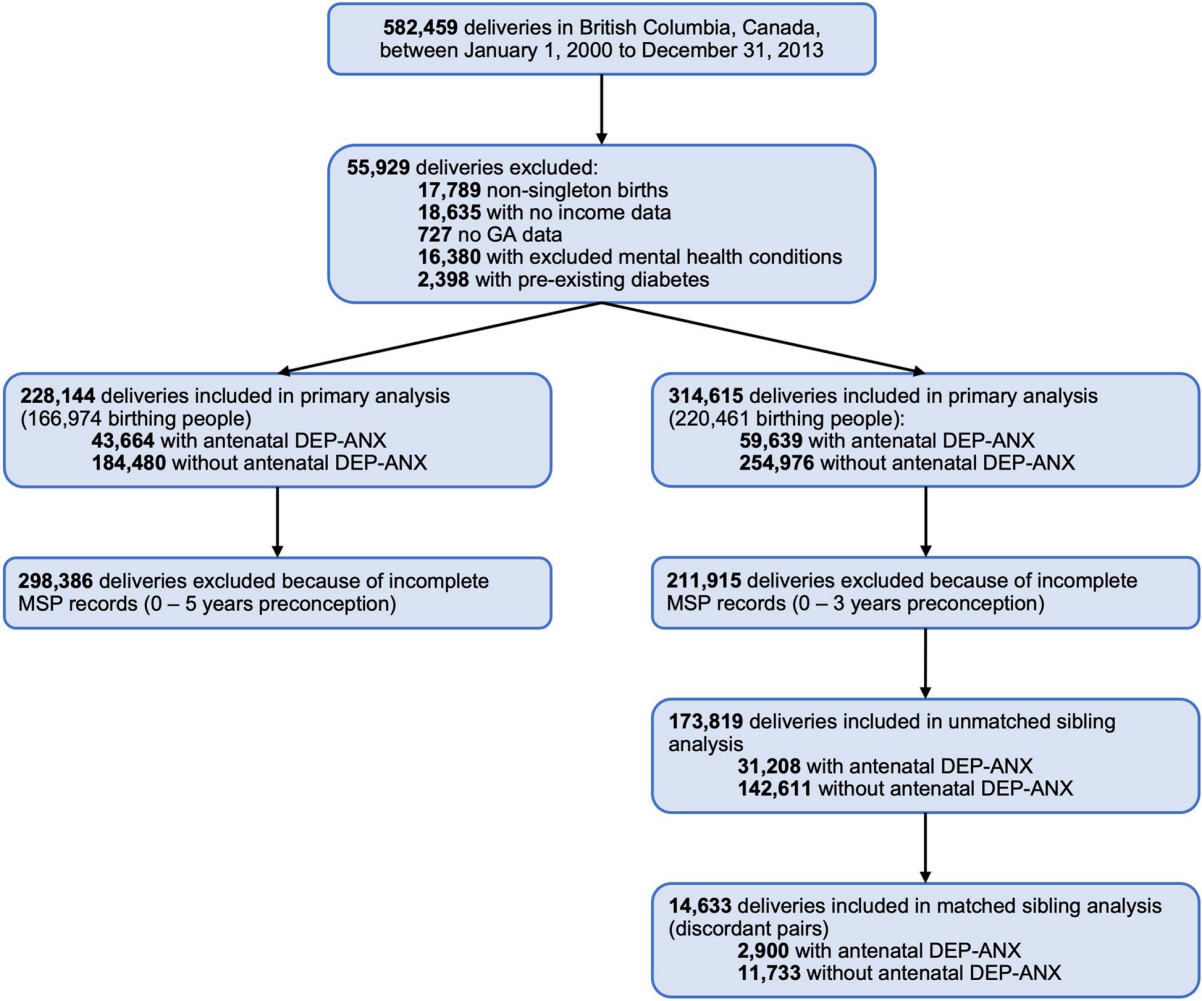
Flow diagram for population-based study in British Columbia, CA, investigating the association between antenatal DEP-ANX and GDM. Abbreviations: DEP-ANX, depression and/or anxiety; GDM, gestational diabetes mellitus

Our cohort included singleton births to birthing people with complete record of neighborhood-based income quintiles and final gestational age (GA). We excluded births to individuals who had pre-existing DM, and/or any record of health service use with diagnostic codes corresponding to bipolar disorder, schizophrenia, psychosis, or mania (Supplemental Table 1). Finally, we required that birthing people be registered with MSP for > 100 days/year from 5-years preconception to birth. We loosened this criterion to 3-years preconception for our sibling cohort analyses, described below, to increase statistical power (see Fig. 1).

**Table 1:** Comparison of socio-demographic and clinical characteristics among individuals with and without antenatal DEP-ANX

	**Antenatal DEP-ANX**		
	**No** N = 184,480	**Yes** N = 43,664	**Standardized difference **^**a**^
**Birth parent socio-demographic factors**			
**Birth parent age group, N (%)**			**0.060**
< 20 years	6136 (3.3)	1610 (3.7)	
20 – 24 years	23,958 (13.0)	5797 (13.3)	
25 – 29 years	48,480 (26.3)	11,021 (25.2)	
30 – 34 years	62,232 (33.7)	14,104 (32.3)	
35 – 39 years	35,735 (19.4)	8858 (20.3)	
≥ 40 years	7939 (4.3)	2274 (5.2)	
**Neighborhood-based income quintile, N (%)**			**0.024**
1	37,931 (20.6)	9215 (21.1)	
2	38,793 (21.0)	9204 (21.1)	
3	38,433 (20.8)	9140 (20.9)	
4	38,792 (21.0)	9238 (21.2)	
5	30,531 (16.5)	6867 (15.7)	
**Marital status, N (%)**			0.145 ^*^
Divorced	2760 (1.5)	926 (2.1)	
Married	128,164 (69.5)	27,473 (62.9)	
Never married	36,243 (19.6)	10,321 (23.6)	
Other	14,493 (7.9)	3925 (9.0)	
Single	2820 (1.5)	1019 (2.3)	
**Coparent status**			
Coparent listed, N (%)	178,246 (96.6)	41,219 (94.4)	0.107 ^*^
Coparent age (years), Mean (SD)	33.5 (6.3)	33.7 (6.6)	0.036
**Number of living children, N (%)**			**0.086**
0	80,664 (43.7)	20,789 (47.6)	
1	69,341 (37.6)	14,748 (33.8)	
2	23,917 (13.0)	5627 (12.9)	
3	6987 (3.8)	1692 (3.9)	
4 or more	3571 (1.9)	808 (1.9)	
**Pregnancy characteristics and risk factors**			
**Year of birth, N (%)**			**0.035**
< 2008	46,504 (25.2)	11,452 (26.2)	
2008 – 2010	67,685 (36.7)	16,298 (37.3)	
> 2010	70,291 (38.1)	15,914 (36.4)	
**Smoking status during pregnancy, N (%)**			0.127 ^*^
No history of smoking	152,610 (82.7)	33,947 (77.7)	
Continued during pregnancy	15,947 (8.6)	5104 (11.7)	
Discontinued during pregnancy	15,923 (8.6)	4613 (10.6)	
**History of premature birth, N (%)**	**7641 (4.1)**	**1983 (4.5)**	**0.020**
**Nulliparous, N (%)**	**79,464 (43.1)**	**20,423 (46.8)**	**0.074**
**Preconception BMI, N (%) **^**b**^			**0.066**
< 18.5 (underweight)	6193 (3.4)	1523 (3.5)	
18.5 – 24.99 (normal)	77,149 (41.8)	18,000 (41.2)	
25.0 – 29.99 (overweight)	28,378 (15.4)	7166 (16.4)	
≥ 30 (obese)	18,167 (9.8)	4948 (11.3)	
Missing	54,593 (29.6)	12,027 (27.5)	
**Gestational diabetes, N (%)**	**15,102 (8.2)**	**4180 (9.6)**	**0.049**
Insulin-dependent	4155 (2.3)	1232 (2.8)	0.036
Non-insulin dependent	10,947 (5.9)	2948 (6.8)	0.034
**Hypertension, N (%)**			
Pregnancy-induced	9167 (5.0)	2560 (5.9)	0.040
Other ^c^	6017 (3.3)	1607 (3.7)	0.023
**Prenatal care, N (%)**			
≥ 10 prenatal visits	58,805 (31.9)	16,148 (37.0)	0.108 ^*^
Prior hospital admission	16,573 (9.0)	5361 (12.3)	0.107 ^*^
**IUGR, N (%)**	**3047 (1.7)**	**815 (1.9)**	**0.016**
**Nature of labor, N (%)**			
Vaginal delivery	129,673 (70.3)	29,538 (67.6)	0.057
Induced labor	37,292 (20.2)	9651 (22.1)	0.046
Midwifery care	27,286 (14.8)	4521 (10.4)	0.134 ^*^
**Postpartum and neonatal characteristics**			
**Infant sex, N (%)**	**89,827 (48.7)**	**21,095 (48.3)**	**0.008**
**Gestational age (weeks), mean (SD)**	**38.7 (1.9)**	**38.6 (2.0)**	**0.067**
**Size at birth **^**d**^			
Small-for-gestational-age, N (%) ^e^	15,701 (8.5)	3858 (8.8)	0.012
Large-for-gestational-age, N (%) ^f^	19,883 (10.8)	4725 (10.8)	0.001
**Admission to NICU, N (%)**	**4454 (2.4)**	**1317 (3.0)**	**0.037**
**Preterm birth, N (%)**	**14,404 (7.8)**	**3978 (9.1)**	**0.047**

Abbreviations: *DEP-ANX* depression and/or anxiety, *BMI* body mass index, *IUGR* intrauterine growth restriction, *NICU* neonatal intensive care unit

^a^ A standardized difference of 0.1 or greater was deemed meaningful and designated with a (*)

^b^ Equal to weight (kilograms) divided by height (meters) squared

^c^ Comprised of preexisting hypertension, high blood pressure, hypertensive kidney disease, proteinuria, HELLP (Hemolysis, Elevated Liver enzymes, and Low Platelets) syndrome, and other hypertensive disorders

^d^ Percentiles determined based on birth weights within gestational age (GA) and infant sex subgroups

^e^ Below the 10^th^ percentile of weight for final GA and sex

^f^ Above the 90^th^ percentile of weight for final GA and sex

### Measures

*Time periods of interest.* We determined approximate date of conception (DOC) by subtracting final GA (reported in the BCPDR) from the offspring’s date of birth (DOB), then subtracting two weeks. Final GA is approximated by the BCPDR based (in order of accuracy) on earliest ultrasound, last menstrual period, or newborn examination. We defined pregnancy as the period between the DOC and DOB, and preconception periods (0 – 1 year, 2 – 3 years, 4 – 5 years, 6 – 10 years, and > 10 years) using the approximate DOC.

**Fig. 2:**
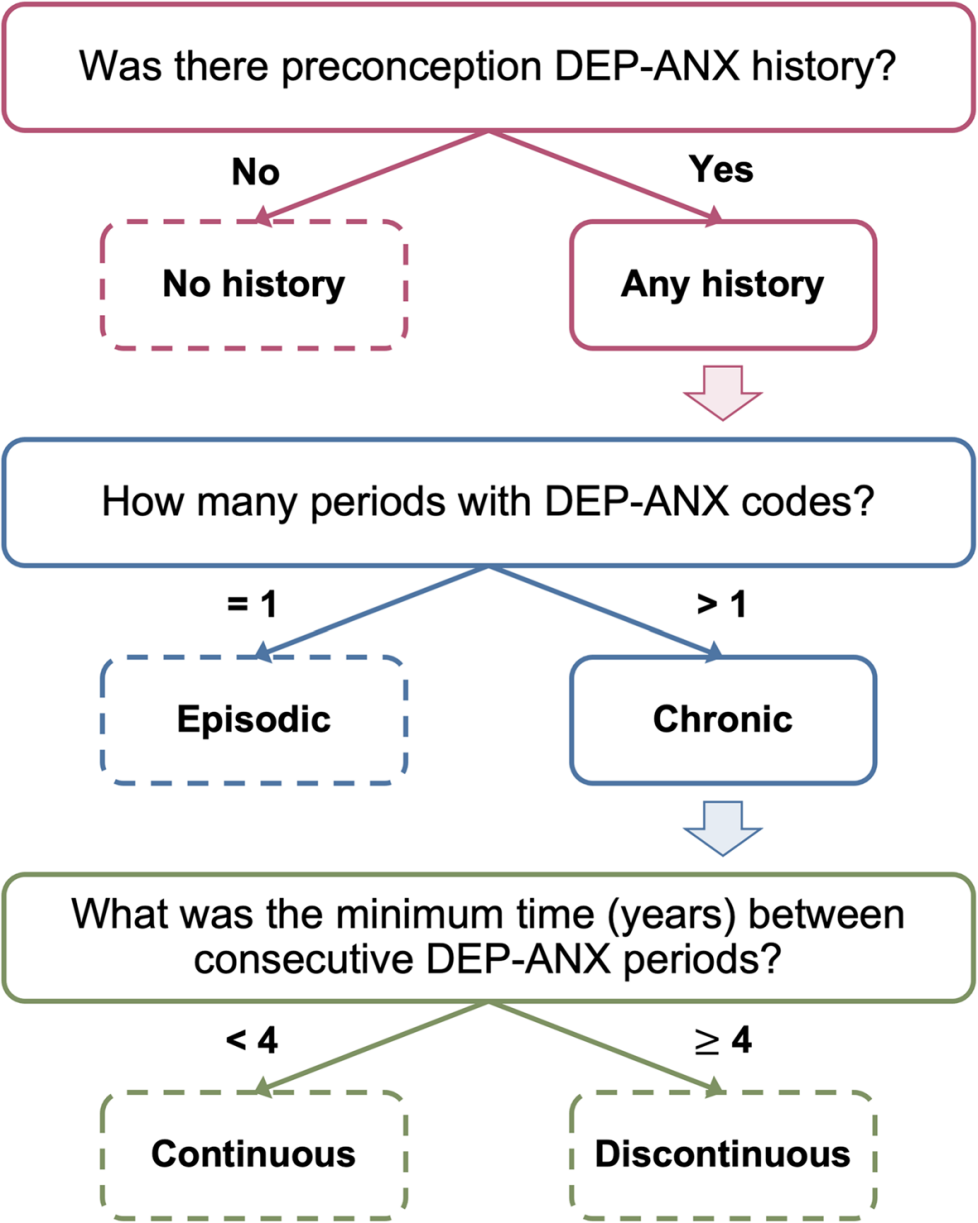
Decision tree for preconception DEP-ANX persistence classification. Abbreviations: DEP-ANX, depression and/or anxiety

*Mental health measures*. Depression and anxiety were classified as a single exposure, DEP-ANX, due to high rates of co-occurrence (particularly within the perinatal period) [40], overlapping risk factors, and neurobiological similarities [41–45]. We identified DEP-ANX cases within each period based on the presence of relevant diagnostic codes (Supplemental Table 2) from fee-for-service provider visits and hospitalization data. MSP outpatient records are coded using the *International Classification of Disease, Ninth Revision, Clinical Modification* (ICD-9-CM), while DAD hospitalizations are coded using the ICD-10-CM. Mental health was further described by DEP-ANX history, independent of antenatal DEP-ANX, according to observed DEP-ANX persistence across preconception. Persistence was categorized into the following groups (Fig. 2): 1) no history; 2) episodic history; 3) chronic history with discontinuous treatment; and 4) chronic history with continuous treatment.

**Table 2:** Association between antenatal DEP-ANX and GDM across preconception DEP-ANX persistence strata. RDs and ORs represent the log likelihood of GDM among those with antenatal DEP-ANX compared to those without

Sample of interest		Frequency of GDM, N (%)		Base model		Adjusted OR (95% CI) ^a^	
**DEP-ANX history**	**Number of deliveries**	**Unexposed**	**Exposed**	**Absolute RD, % (95% CI) **^**a**^	**Unadjusted OR (95% CI) **^**a**^	**Model 1 **^**b**^	**Model 2 **^**c**^
**Base analysis**							
Full cohort	228,144	15,102 (8.2)	4180 (9.6)	1.39 (1.08 – 1.69)	1.19 (1.15 – 1.23)	1.20 (1.15 – 1.24)	1.16 (1.11 – 1.20)
**Stratified analysis**							
No history	91,109	6279 (7.7)	898 (9.3)	1.61 (1.00 – 2.22)	1.23 (1.14 – 1.32)	1.26 (1.17 – 1.36)	1.25 (1.16 – 1.35)
Episodic	62,994	4266 (8.1)	927 (8.9)	0.74 (0.14 – 1.33)	1.10 (1.02 – 1.18)	1.14 (1.06 – 1.23)	1.13 (1.05 – 1.22)
Chronic, discontinuous	7470	506 (9.4)	227 (10.9)	1.45 (-0.10 – 2.99)	1.17 (0.99 – 1.38)	1.21 (1.02 – 1.43)	1.17 (0.98 – 1.39)
Chronic, Continuous	66,571	4051 (9.0)	2128 (9.9)	0.93 (0.45 – 1.41)	1.11 (1.05 – 1.18)	1.13 (1.07 – 1.19)	1.10 (1.04 – 1.17)

Abbreviations: *DEP-ANX* depression and/or anxiety, *GDM* gestational diabetes mellitus. *RD* risk difference, *CI* confidence interval, *OR* odds ratio

^a^ Reference group: no exposure to antenatal DEP-ANX

^b^ Adjusted for year of birth, birth parent age category, neighborhood-based income quintile, marital status, and number of living children

^c^ Adjusted for items in Model 1 and preconception body mass index (BMI), pregnancy-induced hypertension (PIH), non-pregnancy induced hypertension (non-PIH), antenatal hospitalization, and intrauterine growth restriction (IUGR)

### Outcomes

Instances of GDM were identified from the BCPDR based on results of routine prenatal screening (typically between 24 and 28 weeks) [46].

### Covariates

Birthing person and coparent age, coparent status, income quintile, marital status, number of living children, and birth year were used to describe individual-level demographics. Pregnancy health and prenatal care were characterized by smoking status (during the current pregnancy), history of premature birth, parity, preconception body mass index category (BMI; equal to weight [kilograms] divided by height [meters] squared), pregnancy and non-pregnancy induced hypertension (PIH and non-PIH), intrauterine growth restriction (IUGR), frequency of prenatal visits, and frequency of antenatal hospitalization.

We described labor and delivery by use of induction, mode of delivery (vaginal or non-vaginal), and presence of a midwife at birth. Finally, neonatal outcomes were summarized by infant sex, final GA, small-for-gestational-age (SGA; birth weight below 10^th^ percentile for final GA and sex), large-for-gestational-age (LGA; birth weight over 90^th^ percentile for final GA and sex), admission to the neonatal intensive care unit (NICU), and preterm birth.

### Statistical analysis

We compared socio-demographics, health during pregnancy, labor and delivery, and neonatal outcomes between those with and without antenatal DEP-ANX using standardized differences. Differences of 0.1 or more were deemed clinically meaningful [47]. We repeated this process to identify differences in our cohort based on persistence of preconception DEP-ANX.

We modeled the relationship between antenatal DEP-ANX and GDM using logistic regression. Covariates were added in a stepwise fashion to identify potential confounders that significantly improved model fit. Associations between antenatal DEP-ANX exposure and GDM were quantified using absolute risk differences (RDs) and odds ratios (ORs). Unadjusted associations were assessed first (Base Model), followed by adjustment for socio-demographics, including birthing person age category, income quintile, marital status, number of living children, and year of birth (Model 1). Finally, we added characteristics of pregnancy, including preconception BMI, PIH, non-PIH, antenatal hospitalization, and IUGR (Model 2). Regressions were also run following stratification according to persistence of preconception DEP-ANX.

To better adjust for genetic and epigenetic (social and environmental) factors that might confound the relationship between antenatal DEP-ANX and GDM, we conducted analyses in a sibling cohort nested within our larger cohort [48]. This analyzes the association between DEP-ANX and GDM across pregnancies to the same birthing person, rather than comparing across different birthing people. We restricted to birthing people who delivered more than once within the study period and stratified by preconception DEP-ANX history (no history vs. any history). We ran unconditional logistic regression models for the full sibling cohort and each stratum using clustered standard errors to account for correlation between sibling pairs. Associations estimated by these models were used to determine whether the relationship between antenatal DEP-ANX and GDM observed in the full cohort persisted within the sibling cohort. We then ran conditional regression models that matched birthing people to themselves (in successive deliveries), selecting only discordant pregnancies. Only individuals who were discordant in exposure and outcome in ≥ 2 pregnancies contributed to this model.

All *P-*values were two-sided and statistical significance was defined with an alpha of 0.05. All statistical analyses were carried out using RStudio software.

## Results

Between April 1, 2000, and December 31, 2013, there were 582,459 births recorded in BC. Of these, we excluded all multiple births (*N* = 17,789), and births without income (*N* = 18,635) or final GA (*N* = 727) information reported. We further excluded births parents with any health service use for excluded mental health conditions (*N* = 16,380) and birthing people with pre-existing diabetes (*N* = 2,398). Birthing people registered with MSP for < 100 days/year from 5-years preconception to delivery were also excluded (*N* = 298,386). Our final cohort consisted of 228,144 births to 166,974 birthing people. See Supplemental Table 3 for a comparison between included and excluded births.

**Table 3:** Stratified sibling cohort analysis of association between antenatal DEP-ANX and GDM. RDs and ORs represent the risk or log likelihood of GDM among those with antenatal DEP-ANX compared to those without

Sample of interest		Cases of GDM, N (%)		Base model		Adjusted OR (95% CI) ^a^	
**DEP-ANX history**	**Number of deliveries**	**Unexposed**	**Exposed**	**Absolute RD, % (95% CI) **^**a**^	**Unadjusted OR (95% CI) **^**a**^	**Model 1 **^**b**^	**Model 2 **^**c**^
**Base analysis**							
Full cohort	314,615	20,402 (8.0)	5494 (9.2)	1.21 (0.96 – 1.47)	1.17 (1.13 – 1.20)	1.17 (1.14 – 1.22)	1.14 (1.10 – 1.18)
No history	136,416	9353 (7.7)	1350 (9.2)	1.54 (1.04 – 2.03)	1.22 (1.15 – 1.30)	1.24 (1.17 – 1.32)	1.23 (1.16 – 1.31)
Any history	178,199	11,049 (8.3)	4144 (9.2)	0.92 (0.61 – 1.22)	1.12 (1.08 – 1.16)	1.14 (1.10 – 1.19)	1.11 (1.07 – 1.15)
**Unmatched sibling analysis**							
Full cohort	173,819	9842 (6.9)	2507 (8.0)	1.13 (0.80 – 1.46)	1.18 (1.12 – 1.24)	1.20 (1.14 – 1.26)	1.15 (1.09 – 1.21)
No history	72,825	4078 (6.2)	536 (7.5)	1.31 (0.67 – 1.95)	1.23 (1.12 – 1.35)	1.24 (1.17 – 1.32)	1.23 (1.16 – 1.31)
Any history	100,994	5764 (7.5)	1971 (8.2)	0.69 (0.30 – 1.09)	1.10 (1.04 – 1.16)	1.13 (1.07 – 1.20)	1.09 (1.03 – 1.16)
**Matched sibling analysis**							
Full cohort	14,633	5548 (47.3)	1415 (48.8)	1.51 (-0.52 – 3.54)	1.05 (0.96 – 1.15)	1.08 (0.97 – 1.19)	1.07 (0.96 – 1.18)
No history	4726	2116 (46.7)	136 (68.3)	21.60 (14.98 – 28.22)	2.27 (1.68 – 3.07)	1.18 (0.86 – 1.62)	1.18 (0.86 – 1.63)
Any history	7850	2785 (47.8)	977 (48.3)	0.56 (-1.96 – 3.09)	1.01 (0.90 – 1.13)	1.04 (0.91 – 1.18)	1.02 (0.90 – 1.16)

Abbreviations: *DEP-ANX* depression and/or anxiety, *GDM* gestational diabetes mellitus, *RD*, risk difference, *CI* confidence interval *OR* odds ratio

^a^ Reference group: no exposure to antenatal DEP-ANX

^b^ Adjusted for birth parent age category, neighborhood-based income quintile, marital status, number of living children, and birth year

^c^ Adjusted for items in Model 1 and preconception body mass index (BMI), pregnancy-induced hypertension (PIH), non-pregnancy induced hypertension (non-PIH), antenatal hospitalization, and intrauterine growth restriction (IUGR)

Of included births, 43,664 (19.1%) were to individuals with any health service use for DEP-ANX during pregnancy (Table 1). There were 4,180 (9.6%) cases of GDM among those with antenatal exposure to DEP-ANX compared to 15,102 (8.2%) cases among those without antenatal DEP-ANX (SMD 0.049). Compared to those without, those with exposure to antenatal DEP-ANX were less likely to be married (62.9 vs. 69.5%; SMD 0.145), have a coparent present (94.4 vs. 96.6%; SMD 0.107), and/or have midwifery care at delivery (10.4 vs. 14.8%; SMD 0.134). Additionally, birthing people with antenatal DEP-ANX were more likely to smoke during pregnancy (11.7 vs. 8.6%; SMD 0.127), have ≥ 10 prenatal visits (37.0 vs. 31.9%; SMD 0.108), and be hospitalized during pregnancy (12.3 vs. 9.0%; SMD 0.197).

Stratification by preconception DEP-ANX persistence revealed meaningful differences across socio-demographics and pregnancy characteristics, summarized in Supplemental Table 4. The chronic, continuous group was older, less likely to be married and have other living children, and more likely to smoke than those with no DEP-ANX history.

Unadjusted logistic regression models, which examine the relationship across pregnancies to the same birthing person, suggested a significant association between antenatal DEP-ANX and GDM among our full cohort (OR 1.19 [95% CI: 1.15 – 1.23]) as seen in Table 2. This association was maintained after adjusting for birthing person age category, neighborhood-based income quintile, marital status, number of living children, and year of birth (Model 1; aOR 1.20 [95% CI: 1.15 – 1.24]). Following adjustment for preconception BMI, PIH, non-PIH, antenatal hospitalization, and IUGR (Model 2), we saw a slight attenuation in risk for GDM given antenatal DEP-ANX; however, the association remained significant (aOR 1.16 [95% CI: 1.11 – 1.20]).

Stratified analysis revealed differential associations between antenatal DEP-ANX and GDM across persistence groups (Table 2). Unadjusted odds for GDM given antenatal DEP-ANX were highest among those with no DEP-ANX history (OR 1.23 [95% CI: 1.14 – 1.32]) and remained so in the fully adjusted model (Model 2 aOR 1.25 [95% CI 1.16 – 1.35]). Comparatively, the unadjusted association between antenatal DEP-ANX and GDM among those with a chronic, continuous history of DEP-ANX was significantly smaller (OR 1.11 [95% CI: 1.05 – 1.18]). This association was minimally attenuated after adjusting for socio-demographics and pregnancy characteristics (Model 2 aOR 1.10 [95% CI: 1.04 – 1.17]). Among those with an episodic history of DEP-ANX, odds of GDM given antenatal DEP-ANX resembled those among our chronic, continuous group. Associations within our chronic, discontinuous strata were not statistically significant.

To increase statistical power for our stratified sibling analysis, we expanded our cohort to include those registered with MSP < 100 days/year from 3- to 5-years preconception (*N* = 86,471). Our expanded cohort consisted of 314,615 births to 220,461 birthing people. Logistic regression models run with this expanded cohort (Table 3) provided comparable ORs to those from our original cohort (Overall: OR 1.17 [95% CI: 1.13 – 1.20], Model 2 aOR 1.14 [95% CI: 1.10 – 1.18]). Results from our unmatched sibling cohort analysis further demonstrated that restricting this cohort to birthing people with more than 1 eligible pregnancy did not significantly affect the strength or direction of previously observed associations (Overall: OR 1.18 [95% CI: 1.12 – 1.24], Model 2 aOR 1.15 [95% CI: 1.09 – 1.21]).

Unadjusted conditional logistic regression models, which examine the relationship across pregnancies to the same birthing person, suggested substantially different strengths of association (Overall: OR 1.05 [95% CI: 0.96 – 1.15]; No history: OR 2.27 [95% CI: 1.68 – 3.07]; Any history: OR 1.01 [95% CI: 0.90 – 1.13]). Adjusting for socio-demographics and pregnancy characteristics revealed attenuated associations between GDM and antenatal DEP-ANX with loss of statistical significance across the full cohort (Model 2 aOR 1.07 [95% CI: 0.96 – 1.18]), no DEP-ANX history group (Model 2 aOR 1.18 [95% CI: 0.86 – 1.63]), and any DEP-ANX history group (Model 2 aOR 1.02 [95% CI: 0.90 – 1.16); however, the direction of association remained positive in all three cases.

## Comment

### Principal findings

In this population-based, retrospective cohort study, we found a modest association between antenatal DEP-ANX and GDM that differed in effect size based on preconception mental health. Overall, individuals with antenatal DEP-ANX that had no history of DEP-ANX appeared to be at higher risk for GDM than those with an episodic or chronic, continuous history. While these relationships were attenuated, they largely remained statistically significant after adjusting for socio-demographics and pregnancy characteristics in our original and expanded cohort. Matched sibling pairs analysis resulted odds ratios of similar magnitude among those with no DEP-ANX, but the association was no longer statistically significant and thus we cannot rule out residual confounding as an explanation for the associations in the main cohort.

### Results in the context of What is Known

The positive association between DEP-ANX and GDM is consistent with prior literature. Several observational studies have demonstrated significant associations between antenatal DEP-ANX and GDM of varying effect size, reporting increased risks between 52 – 300% from unadjusted and adjusted analysis [11, 22, 28, 49]. In contrast, several studies have suggested that no association exists between GDM and DEP-ANX [20, 25]. Importantly, the magnitudes of these associations are similar to what we have reported (increased risk of 5 – 20%) and their lack of statistical significance may reflect limited statistical power.

### Clinical implications

We observed a slightly larger effect size in the associations between GDM and incident DEP-ANX vs. recurring or chronic DEP-ANX, highlighting the potential role of GDM-induced stress in the development of antenatal DEP-ANX. GDM diagnosis has been previously shown to be a significant stressor, with lasting impacts on the birth person’s physical and emotional well-being [50]. While we cannot rule out residual confounding, an alternative explanation may be that antenatal DEP-ANX and GDM share biological origins (i.e., hypothalamus–pituitary–adrenal (HPA) axis dysregulation and cytokine-mediated inflammatory responses), as has been explored outside the perinatal context [31]. Despite their lower risk for GDM due to antenatal DEP-ANX, significant associations observed for those with preconception DEP-ANX history could also support the hypothesis that allostatic load plays a role in the relationship between GDM and antenatal DEP-ANX. These findings may be explained by a bidirectional mechanism in which development of either condition contributes to development of the other [29, 30].

Regardless of the mechanism connecting antenatal DEP-ANX and GDM, and even in the case of residual confounding, our findings suggest that DEP-ANX and GDM often co-occur and emphasize the importance of ongoing prenatal screening for both GDM and DEP-ANX, particularly among those without a history of either condition. Differences in association based on preconception DEP-ANX persistence may be due to diagnostic bias that delays DEP-ANX treatment among those with discontinuous or no history of DEP-ANX. Further, more regular preconception interactions with mental health services may facilitate easier access to mental health care during pregnancy, thereby mitigating the effects of antenatal DEP-ANX or preventing DEP-ANX recurrence in response to a GDM diagnosis. This points to the importance of addressing barriers to mental health services, particularly during pregnancy, and the value of providing consistent mental health care throughout a birthing person’s life.

### Research implications

Stratification by persistence of preconception DEP-ANX is a novel approach not previously used to understand the relationship between DEP-ANX and GDM. By accounting for variation in preconception DEP-ANX, we were able to report association between mental health history (DEP-ANX) and GDM in a way that sheds light on the potential mechanisms connecting these conditions. Future work is needed to understand how preconception mental health might affect prenatal trajectories of both antenatal DEP-ANX and GDM. Additionally, research that can more precisely explore themes of mental health care access, diagnostic biases, and symptom severity could help elucidate the underlying mechanism linking GDM and DEP-ANX.

### Strengths and limitations

This study is strengthened by its use of population-based administrative datasets and operationalization of more granular definitions for understanding individual DEP-ANX histories. This more nuanced approach allowed for deeper exploration of the relationship between DEP-ANX and GDM. Our study also possessed key limitations. Compared to included individuals, those who were excluded from our study (largely due to not having lived in the province for 5-years preconception) tended to be of lower socioeconomic status with more limited records of overall health status, potentially affecting generalizability of our findings. We did not have access to pharmacy data, prohibiting us from evaluating the role of psychotropic medication in the observed association. Additionally, our definitions for DEP-ANX relied on treatment records filed with provincial health, thus omitting data from individuals seeking care from providers outside BC’s universal health coverage (counselling psychologists, social workers, etc.) and those with under- or un-treated DEP-ANX. Omission of these data may have caused some misclassification of DEP-ANX persistence and/or antenatal DEP-ANX, particularly for those with chronic histories who may have well-established treatment regimens outside of the health system.

Our use of data for 10-years preconception despite applying a registration criterion for 5-years preconception may have also introduced misclassification, specifically for individuals who experienced DEP-ANX prior to 5-years preconception but were not living in the province at that time. This is unlikely to be associated with GDM status, though, and would therefore bias toward the null. Our inability to determine whether GDM preceded antenatal DEP-ANX made interpreting results more challenging. As we do not have date of diagnosis for GDM in the BCPDR and cannot assume that the first DEP-ANX diagnosis code reported in health services data represents actual onset of DEP-ANX, we cannot determine which diagnosis came first. Finally, we cannot rule out the possibility of residual confounding related to both socioeconomic factors that are known to be important determinants of mental health during pregnancy and to yet unmeasured factors that contribute to both GDM and antenatal DEP-ANX.

## Conclusions

Results from this population-based retrospective cohort study suggest an association between antenatal DEP-ANX and GDM that varied based on mental health history. Our analysis could suggest that incident cases of DEP-ANX within pregnancy are more closely associated with GDM compared to recurring or chronic cases. These findings present a novel perspective on the relationship between DEP-ANX and GDM.

## Supplementary Information

**Additional file 1.** 

## Data Availability

Access to data provided by the Data Steward(s) is subject to approval but can be requested for research projects through the Data Steward(s) or their designated service providers.

## Abbreviations

GDM: Gestational diabetes mellitus
DM: Diabetes mellitus
DEP-ANX: Depression/anxiety
BC: British Columbia
PopData BC: Population Data BC
BCPDR: BC Perinatal Data Registry
DAD: Discharge Abstract Database
MSP: Medical Services Plan
GA: Gestational age
DOC: Date of conception
DOB: Date of birth
ICD: International Classification of Diseases
BMI: Body mass index
PIH: Pregnancy induced hypertension
non-PIH: Non-pregnancy induced hypertension
IUGR: Intrauterine growth restriction
SGA: Small-for-gestational-age
LGA: Large-for-gestational-age
NICU: Neonatal intensive care unit
RD: Risk difference
OR: Odds ratio
HPA: Hypothalamus-pituitary-adrenal axis
