# Supplementary material for: Preconception mental health and the relationship between antenatal depression or anxiety and gestational diabetes mellitus: a population-based cohort study
Source: BMC Pregnancy Childbirth. 2022 Aug 31;22:670. doi: 10.1186/s12884-022-05002-5 (PMC9429302; doi:10.1186/s12884-022-05002-5)
Supplement: Supplementary file 1 — Additional file 1. [file 12884_2022_5002_MOESM1_ESM.docx]

**Supplemental Material**

**Title: Preconception mental health and the relationship between antenatal depression or anxiety and gestational diabetes mellitus: A population-based cohort study.**

**Authors:** Ms. Grace A. THIELE^1^, MSc; Deirdre M. RYAN^2^, MD; Tim F. OBERLANDER^3^, MD; Gillian E. HANLEY^1^, PhD

**Affiliations.**

^1^ Department of Gynaecology and Obstetrics, University of British Columbia (UBC), Vancouver, BC, 828 W 10^th^ Ave, Vancouver, BC V5Z 1M9, Canada

^2^ Departments of Psychiatry, University of British Columbia (UBC), 938 W 28^th^ Ave, Vancouver, BC V5Z 4H4, Canada

^3^ Department of Pediatrics, University of British Columbia (UBC), 938 W 28^th^ Ave, Vancouver, BC V5Z 4H4, Canada

**Supplemental Table 1.** List of relevant health conditions and corresponding diagnostic codes. MSP primarily uses the ICD-9-CM system of diagnostic codes, supplemented by a set of BC specific codes (*). DAD uses primarily ICD-10 diagnostic codes.

| **Diagnostic inclusion** | **Descriptor or subtype** | **MSP diagnostic code** | **DAD diagnostic code** |
| --- | --- | --- | --- |
| **Depressive diagnoses** | | | |
| Depressive disorder |  | 311.x | -- |
| Major depressive disorder | Single episode | 296.2 | F32.xx |
|  | Recurrent | 296.3 | F33.xx |
| Persistent depressive disorder | Dysthymic disorders | 300.4x | F34.1, F34.8, F34.9 |
| Psychosis | Depressive type | 298.0x | -- |
| Adjustment disorder or severe stress reaction | Adjustment disorder with depression | 309.0, 309.1 | F43.21 |
|  | Adjustment disorder with depression and anxiety | -- | F43.23 |
| Postpartum mood disturbance |  | 648.44 | F53 |
| Unspecified or other mood disorders |  | 296.9x | F38, F39 |
| **Anxiety diagnoses** | | | |
| Anxiety disorders |  | 300.0x | F41.xx |
| Adjustment disorder or severe stress reaction | Posttraumatic stress disorder | 309.81 | F43.1x |
|  | Adjustment disorder with anxiety | 309.2x | F43.22 |
| Phobic disorders | None | 300.2x | F40.xx |
| Obsessive compulsive disorders |  | 300.3x | F42.xx |
| **Other diagnoses** | | | |
| Depression or anxiety |  | 50B* | -- |

Abbreviations: MSP, Medical Services Plan; ICD, International Classification of Diseases; BC, British Columbia; DAD, Discharge Abstract Database.

**Supplemental Table 2.** Excluded mental health conditions and corresponding diagnostic codes. MSP primarily uses the ICD-9-CM system of diagnostic codes, supplemented by a set of BC specific codes (*). DAD uses primarily ICD-10 diagnostic codes.

| **Diagnostic exclusion** | **Descriptor or subtype** | **MSP diagnostic code** | **DAD diagnostic code** |
| --- | --- | --- | --- |
| Schizophrenia | -- | 295.xx | F20.xx |
|  | Schizotypal disorder | 295.6 | F21.xx |
|  | Schizoaffective disorder | 295.7 | F25.xx |
| Delusional and/or psychotic disorders | -- | 297.xx | F22.xx |
|  | Brief | 293.81, 293.82, 298.3, 298.4, 298.8 | F23.xx |
|  | Shared | 297.3 | F24.xx |
|  | Other | 298.1 | F28.xx |
|  | Unspecified | 298.9 | F29.xx |
| Manic affective disorder | Recurrent episode | 296.1 | F30.xx |
| Bipolar disorders | -- | 296.xx (excluding 296.2, 296.3, and 296.9) | F31.xx |

Abbreviations: MSP, Medical Services Plan; ICD, International Classification of Diseases; BC, British Columbia; DAD, Discharge Abstract Database.

**Supplemental Table 3.** Comparison of socio-demographic factors, pregnancy characteristics and risk factors, and postpartum and neonatal characteristics based on inclusion status. Meaningful differences between covariates were determined using standardized difference. A standardized difference of 0.1 or greater was deemed meaningful and designated with a (*).

|  | **Inclusion status** | | |
| --- | --- | --- | --- |
|  | **Excluded**  N = 354315 | **Included**  N = 228144 | **Standardized mean difference** |
| **Birth parent socio-demographic factors** | | | |
| Birth parent age group, N (%) |  |  | *0.188** |
| < 20 years | 11766 (3.3) | 7746 (3.4) |  |
| 20 – 24 years | 51621 (14.6) | 29755 (13.0) |  |
| 24 – 29 years | 101909 (28.8) | 59501 (26.1) |  |
| 30 – 34 years | 112893 (31.9) | 76336 (33.5) |  |
| 35 – 39 years | 59282 (16.7) | 44593 (19.5) |  |
| ≥ 40 years | 12654 (3.6) | 10213 (4.5) |  |
| Missing | 4190 (1.2) | 0 (0.0) |  |
| Neighborhood income quintile, N (%) |  |  | *0.354** |
| 1 | 80962 (22.9) | 47146 (20.7) |  |
| 2 | 73750 (20.8) | 47997 (21.0) |  |
| 3 | 66285 (18.7) | 47573 (20.9) |  |
| 4 | 61096 (17.2) | 48030 (21.1) |  |
| 5 | 53059 (15.0) | 37398 (16.4) |  |
| Missing | 19163 (5.4) | 0 (0.0) |  |
| Marital status, N (%) |  |  | *0.102** |
| Divorced | 5815 (1.6) | 3686 (1.6) |  |
| Married | 254244 (71.8) | 155637 (68.2) |  |
| Never married | 58466 (16.5) | 46564 (20.4) |  |
| Other | 30327 (8.6) | 18418 (8.1) |  |
| Single | 5463 (1.5) | 3839 (1.7) |  |
| Co-parents |  |  |  |
| Co-parent listed, N (%) | 336645 (95.0) | 219465 (96.2) | 0.058 |
| Co-parent age (years), Mean (SD) | 33.5 (6.4) | 33.5 (6.4) | 0.006 |
| Number of living children, N (%) |  |  | 0.082 |
| 0 | 171491 (48.4) | 101453 (44.5) |  |
| 1 | 124003 (35.0) | 84089 (36.9) |  |
| 2 | 40733 (11.5) | 29544 (12.9) |  |
| 3 | 11829 (3.3) | 8679 (3.8) |  |
| 4 or more | 6259 (1.8) | 4379 (1.9) |  |
| **Pregnancy characteristics and risk factors** | | | |
| Year of birth, N (%) | 2005.0 (3.8) | 2009.5 (2.4) | *1.090** |
| <2008 | 259505 (73.2) | 57956 (25.4) |  |
| 2008 – 2010 | 49021 (13.8) | 83983 (36.8) |  |
| > 2010 | 45789 (12.9) | 86205 (37.8) |  |
| Smoked during pregnancy, N (%) |  |  | *0.124** |
| No history of smoking | 296632 (83.7) | 186557 (81.8) |  |
| Continued during pregnancy | 36986 (10.4) | 21051 (9.2) |  |
| Discontinued during pregnancy | 20697 (5.8) | 20536 (9.0) |  |
| History of premature birth, N (%) | 13746 (3.9) | 9624 (4.2) | 0.017 |
| Nulliparous, N (%) | 168736 (47.6) | 99887 (43.8) | 0.077 |
| Preconception BMI, N (%) ^a^ |  |  | *0.147** |
| < 18.5 (underweight) | 18369 (5.2) | 7716 (3.4) |  |
| 18.5 – 24.99 (normal) | 156720 (44.2) | 95149 (41.7) |  |
| 25.0 – 29.99 (overweight) | 47498 (13.4) | 35544 (15.6) |  |
| ≥30 (obese) | 25965 (7.3) | 23115 (10.1) |  |
| Missing | 105763 (29.8) | 66620 (29.2) |  |
| Gestational diabetes, N (%) | 25762 (7.3) | 19282 (8.5) | 0.044 |
| Insulin-dependent | 6786 (1.9) | 5387 (2.4) | 0.031 |
| Non-insulin dependent | 18976 (5.4) | 13895 (6.1) | 0.032 |
| Hypertension, N (%) |  |  |  |
| Pregnancy-induced | 17738 (5.0) | 11727 (5.1) | 0.006 |
| Other ^b^ | 11890 (3.4) | 7624 (3.3) | 0.001 |
| Prenatal care, N (%) |  |  |  |
| ≥10 prenatal visits | 105050 (29.6) | 74953 (32.9) | 0.069 |
| Prior hospital admissions | 37674 (10.6) | 21934 (9.6) | 0.034 |
| IUGR, N (%) | 9059 (2.6) | 3862 (1.7) | 0.060 |
| Nature of labor, N (%) |  |  |  |
| Vaginal delivery | 247777 (69.9) | 159211 (69.8) | 0.003 |
| Induced labor | 74733 (21.1) | 46943 (20.6) | 0.013 |
| Midwifery care | 27379 (7.7) | 31807 (13.9) | *0.201** |
| Antenatal DEP-ANX, N (%) | 63342 (17.9) | 43664 (19.1) | 0.032 |
| **Postpartum and neonatal characteristics** | | | |
| Infant sex, N (%) | 172574 (48.7) | 110922 (48.6) | 0.002 |
| Gestational age (weeks), mean (SD) | 38.6 (2.8) | 38.7 (1.9) | 0.055 |
| Size at birth ^c^ |  |  |  |
| Small-for-gestational-age, N (%) ^d^ | 38696 (10.9) | 19559 (8.6) | 0.079 |
| Large-for-gestational-age, N (%) ^e^ | 33588 (9.5) | 24608 (10.8) | 0.043 |
| Admission to NICU, N (%) | 6025 (1.7) | 5771 (2.5) | 0.058 |
| Preterm birth, N (%) | 36429 (10.3) | 18382 (8.1) | 0.077 |

Abbreviations: BMI, body mass index; IUGR, intrauterine growth restriction; DEP-ANX, depression and/or anxiety; NICU, neonatal intensive care unit.

^a^ Equal to weight (kilograms) divided by height (meters) squared.

^b^ Comprised of preexisting hypertension, high blood pressure, hypertensive kidney disease, proteinuria, HELLP (Hemolysis, Elevated Liver enzymes, and Low Platelets) syndrome, and other hypertensive disorders.

^c^ Percentiles determined based on birth weights within gestational age (GA) and infant sex subgroups.

^d^ Below the 10^th^ percentile of weight for final GA and sex.

^e^ Above the 90^th^ percentile of weight for final GA and sex.

**Supplemental Table 4.** Comparison of socio-demographic factors, pregnancy characteristics and risk factors, and postpartum and neonatal characteristics based on persistence of preconception DEP-ANX. Meaningful differences between covariates were determined using standardized difference. A standardized difference of 0.1 or greater was deemed meaningful and designated with a (*).

|  | **Persistence of DEP-ANX prior to pregnancy** | | | | |
| --- | --- | --- | --- | --- | --- |
|  | **No history**  N = 91109 | **Episodic**  N = 62994 | **Discontinuous**  N = 7470 | **Continuous**  N = 66571 | **Standardized difference** |
| **Birth parent socio-demographic factors** | | | | | |
| Birth parent age group, N (%) |  |  |  |  | *0.177** |
| < 20 years | 4228 (4.6) | 2012 (3.2) | 95 (1.3) | 1411 (2.1) |  |
| 20 – 24 years | 12918 (14.2) | 8144 (12.9) | 608 (8.1) | 8085 (12.1) |  |
| 25 – 29 years | 23917 (26.3) | 16430 (26.1) | 1864 (25.0) | 17290 (26.0) |  |
| 30 – 34 years | 30204 (33.2) | 21260 (33.7) | 2764 (37.0) | 22108 (33.2) |  |
| 35 – 39 years | 16303 (17.9) | 12364 (19.6) | 1742 (23.3) | 14184 (21.3) |  |
| ≥ 40 years | 3539 (3.9) | 2784 (4.4) | 397 (5.3) | 3493 (5.2) |  |
| Income quintile, N (%) |  |  |  |  | 0.028 |
| 1 | 18717 (20.5) | 12868 (20.4) | 1504 (20.1) | 14057 (21.1) |  |
| 2 | 19073 (20.9) | 13286 (21.1) | 1578 (21.1) | 14060 (21.1) |  |
| 3 | 19035 (20.9) | 13227 (21.0) | 1485 (19.9) | 13826 (20.8) |  |
| 4 | 19139 (21.0) | 13230 (21.0) | 1668 (22.3) | 13993 (21.0) |  |
| 5 | 15145 (16.6) | 10383 (16.5) | 1235 (16.5) | 10635 (16.0) |  |
| Marital status, N (%) |  |  |  |  | *0.135** |
| Divorced | 981 (1.1) | 950 (1.5) | 161 (2.2) | 1594 (2.4) |  |
| Married | 65040 (71.4) | 43634 (69.3) | 4881 (65.3) | 42082 (63.2) |  |
| Never married | 17274 (19.0) | 12453 (19.8) | 1716 (23.0) | 15121 (22.7) |  |
| Other | 6842 (7.5) | 4972 (7.9) | 516 (6.9) | 6088 (9.1) |  |
| Single | 972 (1.1) | 985 (1.6) | 196 (2.6) | 1686 (2.5) |  |
| Co-parents |  |  |  |  |  |
| Co-parent listed, N (%) | 88282 (96.9) | 60836 (96.6) | 7116 (95.3) | 63231 (95.0) | 0.060 |
| Co-parent age (years), Mean (SD) | 33.0 (6.4) | 33.6 (6.3) | 34.5 (6.1) | 34.1 (6.4) | *0.129** |
| Living children, N (%) |  |  |  |  | *0.176** |
| 0 | 46622 (51.2) | 27186 (43.2) | 2975 (39.8) | 24670 (37.1) |  |
| 1 | 31073 (34.1) | 24161 (38.4) | 2860 (38.3) | 25995 (39.0) |  |
| 2 | 9554 (10.5) | 8173 (13.0) | 1085 (14.5) | 10732 (16.1) |  |
| 3 | 2523 (2.8) | 2340 (3.7) | 345 (4.6) | 3471 (5.2) |  |
| 4 or more | 1337 (1.5) | 1134 (1.8) | 205 (2.7) | 1703 (2.6) |  |
| **Pregnancy characteristics and risk factors** | | | | | |
| Year of birth, N (%) |  |  |  |  | *0.253** |
| < 2008 | 24714 (27.1) | 16082 (25.5) | 823 (11.0) | 16337 (24.5) |  |
| 2008 - 2010 | 33226 (36.5) | 23443 (37.2) | 2408 (32.2) | 24906 (37.4) |  |
| > 2010 | 33169 (36.4) | 23469 (37.3) | 4239 (56.7) | 25328 (38.0) |  |
| Smoking status, N (%) |  |  |  |  | *0.164** |
| No history | 78214 (85.8) | 51888 (82.4) | 5694 (76.3) | 50761 (76.3) |  |
| Continued during pregnancy | 6085 (6.7) | 5652 (9.0) | 824 (10.9) | 8490 (12.8) |  |
| Discontinued during pregnancy | 6810 (7.5) | 5454 (8.7) | 993 (12.8) | 7320 (11.0) |  |
| Nulliparous, N (%) | 46121 (50.6) | 26729 (42.4) | 2906 (39.9) | 24131 (36.2) | *0.158** |
| History of premature birth, N (%) | 2807 (3.1) | 2602 (4.1) | 389 (5.2) | 3826 (5.7) | 0.074 |
| Preconception BMI, N (%) ^a^ |  |  |  |  | 0.086 |
| < 18.5 (underweight) | 3234 (3.5) | 2180 (3.5) | 242 (3.2) | 2060 (3.1) |  |
| 18.5 – 24.99 (normal) | 39714 (43.6) | 26623 (42.4) | 2977 (39.9) | 25835 (38.8) |  |
| 25.0 – 29.99 (overweight) | 13637 (15.0) | 9653 (15.3) | 1249 (16.7) | 11005 (16.5) |  |
| ≥30 (obese) | 7961 (8.7) | 6181 (9.8) | 888 (11.9) | 8085 (12.1) |  |
| Missing | 26563 (29.2) | 18357 (29.1) | 2114 (28.3) | 19586 (29.4) |  |
| Gestational diabetes, N (%) | 7177 (7.9) | 5193 (8.2) | 733 (9.8) | 6179 (9.3) | 0.040 |
| Insulin-dependent | 1763 (1.9) | 1387 (2.2) | 242 (3.2) | 1995 (3.0) | 0.050 |
| Non-insulin dependent | 5414 (5.9) | 3806 (6.0) | 491 (6.6) | 4184 (6.3) | 0.015 |
| Hypertension, N (%) |  |  |  |  |  |
| Pregnancy-induced | 4635 (5.9) | 3081 (4.9) | 382 (5.1) | 3629 (5.5) | 0.013 |
| Other ^b^ | 2971 (3.3) | 2010 (3.2) | 274 (3.7) | 2369 (3.6) | 0.016 |
| Prenatal care, N (%) |  |  |  |  |  |
| ≥10 prenatal visits | 28323 (31.1) | 20588 (32.7) | 2670 (35.7) | 23372 (35.1) | 0.058 |
| Prior hospital admissions | 7384 (8.1) | 5782 (9.2) | 792 (10.6) | 7976 (12.0) | 0.073 |
| IUGR, N (%) | 1497 (1.6) | 1058 (1.7) | 123 (1.6) | 1184 (1.8) | 0.006 |
| Nature of labor, N (%) |  |  |  |  |  |
| Vaginal delivery | 65068 (71.4) | 44045 (69.9) | 4945 (66.2) | 45153 (67.8) | 0.064 |
| Induced labor | 18359 (20.2) | 12931 (20.5) | 1567 (21.0) | 14086 (21.2) | 0.014 |
| Midwifery care | 12695 (13.9) | 8976 (14.2) | 1157 (15.5) | 8979 (13.5) | 0.030 |
| Antenatal mental health service use |  |  |  |  |  |
| DEP-ANX diagnosis, N (%) | 9635 (10.6) | 10466 (16.6) | 2091 (28.0) | 21472 (32.3) | *0.319** |
| Frequency of outpatient visits for DEP-ANX, N (%) |  |  |  |  | *0.371** |
| Low | 35034 (38.5) | 21848 (34.7) | 1127 (15.1) | 18039 (27.1) |  |
| Moderate | 32757 (36.0) | 21632 (34.3) | 2734 (36.6) | 18925 (28.4) |  |
| High | 23318 (25.6) | 19514 (31.0) | 3609 (48.3) | 29607 (44.5) |  |
| Use of a psychiatrist for DEP-ANX, N (%) | 336 (0.4) | 618 (1.0) | 274 (3.7) | 3278 (4.9) | *0.179** |
| **Postpartum and neonatal characteristics** | | | | | |
| Infant sex = female, N (%) | 44368 (48.7) | 30535 (48.5) | 3666 (49.1) | 32353 (48.6) | 0.005 |
| Gestational age (weeks), mean (SD) | 38.8 (1.9) | 38.7 (1.9) | 38.5 (2.1) | 38.5 (2.0) | 0.083 |
| Size at birth ^c^ |  |  |  |  |  |
| Small-for-gestational-age, N (%) ^d^ | 8296 (9.1) | 5301 (8.4) | 602 (8.1) | 5360 (8.1) | 0.021 |
| Large-for-gestational-age, N (%) ^e^ | 9098 (10.0) | 6827 (10.8) | 847 (11.3) | 7836 (11.8) | 0.031 |
| Admission to NICU, N (%) | 2263 (2.5) | 1479 (2.3) | 176 (2.4) | 1853 (2.8) | 0.015 |
| Preterm birth, N (%) | 6627 (7.3) | 4937 (7.8) | 698 (9.3) | 6120 (9.2) | 0.046 |

Abbreviations: DEP-ANX, depression and/or anxiety; BMI, body mass index; IUGR, intrauterine growth restriction; DEP-ANX, depression and/or anxiety; NICU, neonatal intensive care unit.

^a^ Equal to weight (kilograms) divided by height (meters) squared.

^b^ Comprised of preexisting hypertension, high blood pressure, hypertensive kidney disease, proteinuria, HELLP (Hemolysis, Elevated Liver enzymes, and Low Platelets) syndrome, and other hypertensive disorders.

^c^ Percentiles determined based on birth weights within gestational age (GA) and infant sex subgroups.

^d^ Below the 10^th^ percentile of weight for final GA and sex.

^e^ Above the 90^th^ percentile of weight for final GA and sex.
